# Genome-Wide Identification and Expression Profiling of Plasma Membrane-Localized SWEET Gene Family Associated with Sugar Transport During Yam Tuber Development

**DOI:** 10.3390/ijms26125847

**Published:** 2025-06-18

**Authors:** Na Li, Yanfang Zhang, Xiuwen Huo, Linan Xing, Mingran Ge, Ningning Suo

**Affiliations:** College of Horticulture and Plant Protection, Inner Mongolia Agricultural University, Hohhot 010018, China; lsf15048456237@163.com (N.L.); zhangyanfang@imau.edu.cn (Y.Z.); xln620719@163.com (L.X.); gemingran1996@163.com (M.G.); suoning200@gmail.com (N.S.)

**Keywords:** yam, SWEET, yeast substrate assays, gene expression

## Abstract

This study provides the first comprehensive genome-wide identification and characterization of the *SWEET* gene family in yam (*Dioscorea rotundata*), integrating structural bioinformatics, gene expression profiling, and functional validation to explore its roles in sucrose transport and tuber development. A total of 19 *SWEET* genes were identified and predicted to localize to the plasma membrane, and they showed high phylogenetic conservation with *Arabidopsis thaliana*, suggesting conserved functions in sugar distribution. Yeast substrate assays revealed that *DrSWEET6* and *DrSWEET12* are capable of transporting both hexose and sucrose across the plasma membrane, with their expression predominantly observed in the tuber, implicating their involvement in sucrose unloading. Expression profiling indicated high expression levels of the *SWEET* genes at the tuber apex, which progressively increased during tuber development, underscoring their critical roles in sucrose unloading, cell expansion, and biomass accumulation. These findings provide novel insights into the structural and functional mechanisms of the SWEET-mediated sucrose transport in yam, laying a solid foundation for future crop improvement strategies aiming to optimize sucrose distribution and enhance tuber yield and quality.

## 1. Introduction

Sucrose is synthesized in the leaves of plants and transported through the phloem to vascular tissue for long-distance distribution. SWEET is the final export transporter responsible for effluxing sugars from the plant cell, and it facilitates sugar loading into the phloem through sugar transporters (SUTs) [1,2]. Sugars are then transferred from the phloem to sink tissues, including fruits, tubers, roots, and seeds, where they are stored or utilized [3,4]. In particular, sucrose and other soluble sugars play essential roles in starch granule formation in storage roots [5]. Consequently, changes in how sugars are distributed within the plant can significantly influence starch accumulation.

SWEET (Sugars Will Eventually be Exported Transporters) proteins represent a newly identified class of sugar transporters that are widely distributed across the plant kingdom. These proteins play a critical role not only in the metabolism and transport of sugars within plants but also in their responses to environmental stressors. The SWEET family encompasses a distinctive group of sugar transport proteins that facilitate the bidirectional transport of sucrose and hexoses in both plants and animals [6]. Plant SWEET proteins typically consist of seven transmembrane domains, characterized by two MtN3/saliva (PF03083) motifs located within the cell membrane [7,8].SWEET family members of various evolutionary branches exhibit preferences for transporting different types of sugars. These preferences include hexoses (branches I and II), sucrose (branch III), and fructose (branch IV) [9,10,11]. For example, class III includes the *Arabidopsis SWEET* genes *AtSWEET*9-15, which are involved in sucrose transport and play a critical role in starch accumulation [12]. Sugar transporters are central to sugar translocation, regulated by various mechanisms, including transcription factor modulation and post-translational modifications. They are crucial for numerous physiological processes, such as plant defense, long-distance sucrose transport, nutritional and reproductive growth, senescence, and stress responses [13,14,15]. Research on SWEET proteins in different plant species has provided valuable insights into their diverse functions. In tomato fruits, *SlSWEET7a* and *SlSWEET14* negatively regulate sugar transport and storage, while in apples, *MdSWEET9b* promotes sugar accumulation, shedding light on hormone–sugar metabolic interactions [16]. In soybeans, *GmSWEET15* mediates sucrose export from the endosperm to developing embryos, illustrating the protein’s role in seed development. Additionally, in tea plants, *CsSWEET17* enhances cold tolerance by interacting with CsLHY [17].In pineapple, *SWEET10* has been identified as a potential glucose transporter that could improve fruit yield and quality by modulating the transport activity of *AcSWEET10* [18].

Yam, a nutrient-rich tuber, is widely cultivated in Asia, Africa, and the Americas, serving as a major carbohydrate source [19]. Despite its nutritional and agricultural importance, molecular studies on yam are limited, with most focusing on nutritional composition analysis. In particular, the mechanisms of carbohydrate transport during tuber development, especially those involving sucrose translocation, remain poorly understood. While previous studies in other crops have largely focused on gene identification and expression profiling, our study integrates bioinformatics analysis and functional assays to uncover conserved substrate-binding features in yam SWEET proteins, offering novel insights into transporter engineering. Given the key role of sucrose transporters in biomass accumulation and sink organ development, we performed a genome-wide analysis of the *SWEET* gene family in yam, including phylogenetic relationships, gene structures, and expression profiles across developmental stages. Furthermore, we identified two SWEET proteins, DrSWEET6 and DrSWEET12, with expression patterns linked to key stages of tuber development and validated their substrate specificity through yeast uptake assays. Growth assays in yeast mutant strains further demonstrated that these transporters affect sucrose-dependent growth, confirming their physiological relevance. Together, our results bridge genomic, bioinformatics, and functional analyses to provide new insights into SWEET-mediated carbohydrate transport in yam. This work not only enhances the current understanding of tuber sink strength and sugar allocation but also lays the groundwork for molecular breeding strategies to improve yield and quality in yam and other tuber crops.

## 2. Results

### 2.1. Identification of Members of the SWEET Gene Family

The *DrSWEET* gene family was identified in the *Dioscorea* genome using HMM (hidden Markov model) searches. Initially, 13 *DrSWEET* genes were obtained, and further analysis using the SMART database was conducted to filter out incomplete sequences. As a result, a total of 19 *DrSWEET* genes were identified and designated as *DrSWEET*1 to *DrSWEET*19 (Table 1). The DrSWEET protein sequences exhibited varying lengths, ranging from 151 amino acids (DrSWEET10) to 300 amino acids (DrSWEET14), with an average length of 322 amino acids. Their molecular weights (MWs) ranged from 16.77 kDa (DrSWEET10) to 33.68 kDa (DrSWEET14). The isoelectric point (pI) values varied between 5.58 (DrSWEET10) and 9.63 (DrSWEET6), with an average of 8.8. The instability index ranged from 27.54 to 49.59, suggesting varying degrees of protein stability. None of the DrSWEET proteins contained signal peptides, and all were classified as hydrophobic proteins, except for DrSWEET2 and DrSWEET6 (Supplementary Table S3). Secondary structure predictions revealed that the DrSWEET proteins were predominantly composed of random coils (29.77–54.55%), followed by α-helices (24.65–42.82%), extended strands (14.32–20.05%), and β-turns (4.88–8.15%) (Supplementary Table S1). Furthermore, all 19 DrSWEET proteins were predicted to be localized in the cell membrane, suggesting that the *SWEET* gene family primarily functions in membrane-associated regulatory processes.

### 2.2. Phylogenetic Analysis and Classification of DrSWEET Proteins

**Figure 1:**
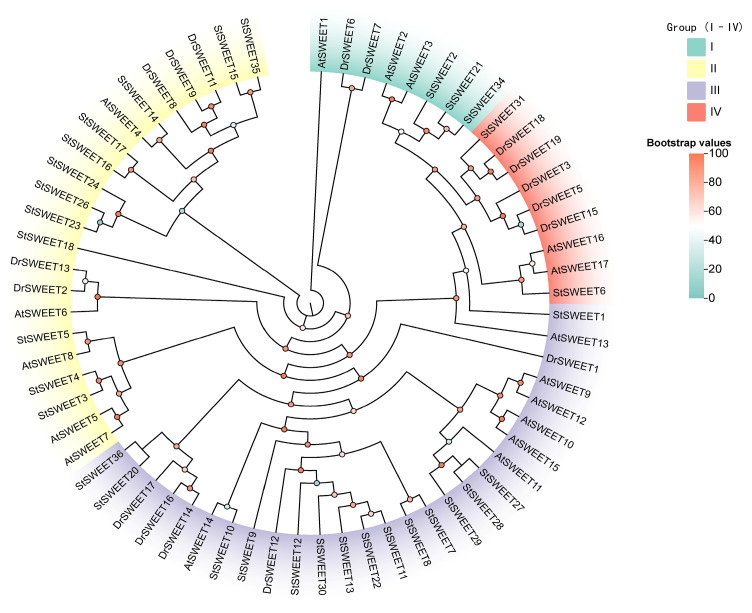
Phylogenetic tree of SWEET proteins in *Arabidopsis thaliana*, *Dioscorea rotundata*, and Solanum tuberosum. The tree was constructed using the conserved MtN3/saliva domain (residues ~60–180). Multiple sequence alignment was performed with MAFFT v7, and the phylogenetic tree was inferred using the maximum likelihood method in IQ-TREE with automatic model selection (ModelFinder). Branch support was assessed using 1000 ultrafast bootstrap replicates. Bootstrap values are represented by gradient-filled circles, with darker shades indicating higher bootstrap support (ranging from 0 to 100; see legend). SWEET proteins were classified into four groups (groups I–IV) based on the AtSWEET reference, and other species were assigned according to their phylogenetic proximity. Group colors are used to indicate classification.

A phylogenetic tree was constructed based on 36 identified DrSWEET proteins and their homologs in the model plant *Arabidopsis thaliana* and the tuber crop Solanum tuberosum. These proteins were classified into four subfamilies (I–IV) according to sequence similarity (Figure 1). Subfamily I comprised *DrSWEET6* and *DrSWEET7*, clustering together with *AtSWEET1*, *AtSWEET2*, *AtSWEET3*, and *StSWEET2*.Subfamily II comprised a smaller number of members, including *DrSWEET18* and *DrSWEET19*, which clustered with *AtSWEET5* and *AtSWEET7*, indicating a distinct lineage. Subfamily III contained *DrSWEET12*, *DrSWEET14*, *DrSWEET16*, and *DrSWEET17*, as well as several potato SWEET proteins (e.g., *StSWEET9* and *StSWEET10*), which were closely related to the Arabidopsis clade III members *AtSWEET10–15* and multiple *StSWEET* homologs, suggesting a relatively conserved evolutionary trajectory. Subfamily IV included *DrSWEET3*, *DrSWEET5*, and *DrSWEET15*, along with *AtSWEET16* and *AtSWEET17*. It is noteworthy that the functions of the SWEET proteins in *Arabidopsis* have been well characterized, with established roles in sugar transport, plant development, and stress responses. Thus, *Arabidopsis* SWEETs serve as valuable references for functional inference. In addition, the inclusion of the *SWEET* homologs in potato, a representative tuber crop, facilitates comparative analyses and highlights the evolutionary conservation and diversification of the *SWEET* gene family across different plant types. These findings provide a theoretical basis for future functional characterization and potential applications in crop improvement.

### 2.3. Chromosome Distribution, Duplication Events, and Collinear Analysis of DrSWEET Genes

**Figure 2:**
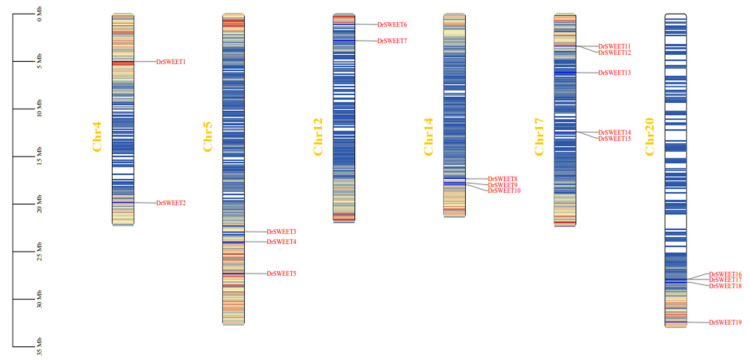
Chromosomal locations of *SWEET* genes. The scale on the left was used to estimate the length of the chromosomes. The *SWEET* genes of *Dioscorea rotundata* were distributed on four chromosomes.

A chromosomal localization analysis showed that the 19 *DrSWEET* genes mapped to six chromosomes, with gene numbers ranging from 2 to 5 per chromosome (Figure 2). Chromosome 17 contained the highest number of *DrSWEET* genes (five; *DrSWEET*11–*DrSWEET*15), accounting for 26.32% of the total. Chromosome 20 harbored four genes (*DrSWEET*16–*DrSWEET*19), representing 21.05% of the family. The fewest genes were found on chromosomes 4 and 12, each containing two genes (*DrSWEET*1–*DrSWEET*2 and *DrSWEET*6–*DrSWEET*7, respectively), representing 10.53% of the total. Additionally, the clustering of *DrSWEET* genes on chromosomes 5 and 14 might result from gene duplication events, which could contribute to the expansion of the gene family and functional diversification.

### 2.4. Evolutionary Analysis of DrSWEET Genes and Their Expansion in Several Different Species

**Figure 3:**
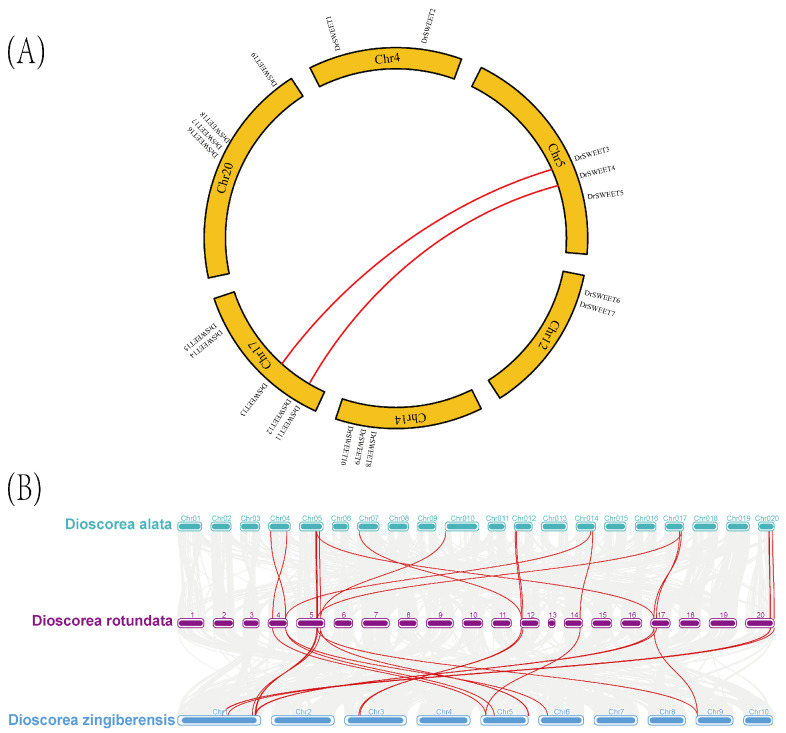
(**A**) Collinearity analysis of *SWEET* genes in *Dioscorea rotundata*. The *DrSWEET* genes are localized on different chromosomes. Chromosome numbers are indicated in the yellow box. The numbers in the chromosome boxes represent the sequence length in megabases. Gene pairs with sibling relationships are connected by a red line. (**B**) Collinearity analysis map of *Dioscorea rotundata* with *Dioscorea* alata and *Dioscorea* zingiberensis, with the red line indicating collinear gene pairs.

Gene duplication is a major factor influencing the formation, expansion, and functional diversification of gene families. To better understand the gene duplication events among *DrSWEET* family members, we analyzed duplication events among the 19 *DrSWEET* genes in the *Dioscorea* genome. A total of two pairs of collinear genes, comprising four duplicated genes (21.05%), were identified and mapped across six chromosomes. Specifically, duplicated genes were located on chromosomes 5 (two clusters) and 17 (two clusters). These two pairs of duplicated genes exhibited high sequence homology (Figure 3A), and all were classified as segmental duplications, indicating that the *DrSWEET* gene family likely expanded through gene duplication events. The presence of segmentally duplicated genes suggests that segmental duplication played a crucial role in the expansion of the *DrSWEET* gene family and was a major driver of *DrSWEET* gene evolution. Notably, although multiple segmental duplications were detected during the expansion of the *DrSWEET* gene family, no tandem duplications were observed. This suggests that the *DrSWEET* genes expanded independently of closely linked neighboring genes, with limited gene interference in adjacent regions. A comparative synteny analysis revealed that eight *DrSWEET* genes exhibited synteny with *Dioscorea rotundata*, followed by *Dioscorea zingiberensis* and *Dioscorea alata*. A homology analysis further confirmed that the *DrSWEET* genes shared 13 syntenic genes with *Dioscorea zingiberensis* and 16 syntenic genes with *Dioscorea alata*, indicating a high degree of conservation and evolutionary relevance among these *Dioscorea* species. The evolutionary relationships and collinearity of the *DrSWEET* genes in *Dioscorea rotundata* with those in *Dioscorea alata* and *Dioscorea zingiberensis* were analyzed. According to the results shown in the figure, a number of collinear gene pairs were identified among the three *Dioscorea* species, as indicated by the red lines. Among them, *Dioscorea rotundata* showed the highest number of collinear pairs with D. alata, indicating a closer genetic relationship between these two species; this suggests that these genes may have played significant roles in plant evolution and may be functionally conserved across diverse plant lineages. These findings highlight the evolutionary conservation and functional importance of *DrSWEET* genes, providing insights into their potential regulatory roles in *Dioscorea* and related plant species (Figure 3B).

### 2.5. Gene Structure and Motif Composition of the DrSWEET Genes

**Figure 4:**
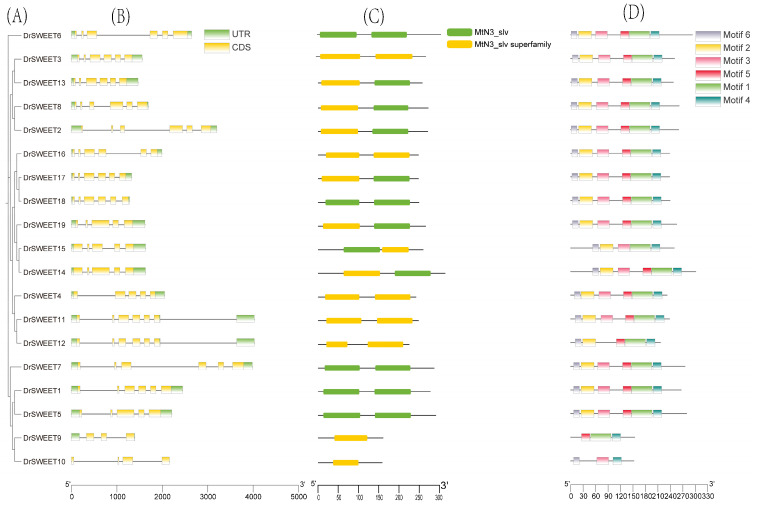
Conserved motif and gene structure analysis of the *SWEET* gene family in *Dioscorea rotundata*. (**A**) Phylogenetic tree constructed using the neighbor-joining (NJ) method in MEGA 11. (**B**) Exon–intron structure of *SWEET* genes. (**C**) Schematic representation of conserved domains. (**D**) Conserved motifs of the *SWEET* gene family, represented by colored boxes. The three analyses were integrated using Gene Structure View in TBtools software (v2.310).

To further explore the structural diversity of the *DrSWEET* gene family, exon–intron organization was visualized based on genome annotation data. Most *DrSWEET* genes contained 3–6 exons and 2–5 introns, with the exception of *DrSWEET*10, which exhibited a markedly reduced structure—comprising only 4 exons and 3 introns—due to an extensive loss of conserved domains. Notably, *DrSWEET*10 lacked untranslated regions (UTRs), whereas *DrSWEET*16, *DrSWEET*17, and *DrSWEET*18 harbored relatively short 5’ UTRs. Approximately 63.16% of the *DrSWEET* genes contained six exons and five introns, suggesting a high degree of structural conservation. Despite variation in exon and intron lengths, the overall exon–intron arrangement within each subfamily was largely conserved (Figure 4A,B). To further characterize the structural features and potential functions of *DrSWEET* genes, their conserved motif compositions were analyzed alongside their gene structures. Six conserved motifs (motifs 1–6) were identified, with each protein containing at least three motifs and up to six, primarily located in the N-terminal region. Proteins with similar motif patterns tended to cluster together phylogenetically, suggesting potential functional similarity within subgroups. With the exception of DrSWEET9 and DrSWEET10, most members contained three or more conserved motifs. Motifs 2, 3, and 6 were frequently observed; they are considered functionally critical, as they often contain MtN3_slv repeat domains, which enhance internal sequence conservation (Figure 4C,D). Motif 1 was present in all DrSWEET proteins, except for DrSWEET10, while motif 5 was absent in DrSWEET10 and DrSWEET15, and motif 3 was missing in DrSWEET9 and DrSWEET12. Notably, DrSWEET14 and DrSWEET15 exhibited motif distributions distinct to those of other members, highlighting structural divergence across subfamilies. Overall, *DrSWEET* genes within the same subfamily generally displayed similar motif compositions, supporting the hypothesis of conserved functional roles.

### 2.6. Cis-Regulatory Elements in the Promoters

**Figure 5:**
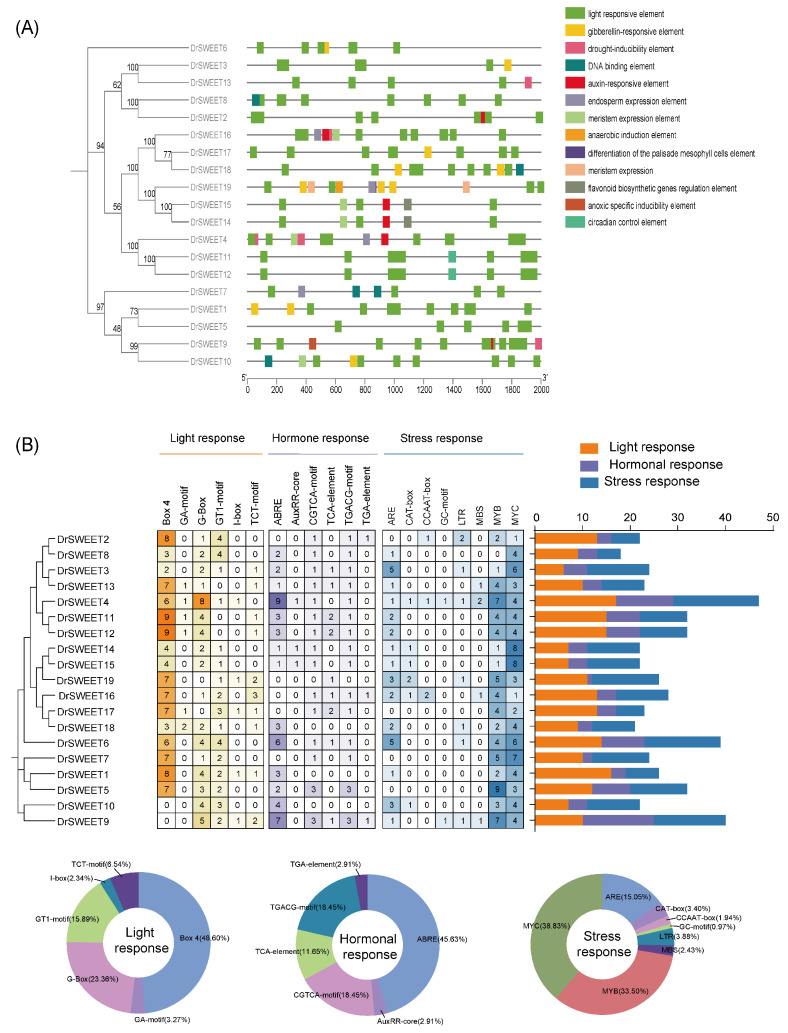
The cis-regulatory elements involved in phytohormone, development, and stress responses in the upstream regions of *DrSWEET* gene promoters. (**A**) Analysis of the positional distribution of cis-regulatory elements. (**B**) Statistical analysis of cis-regulatory elements. ARE, involved in anaerobic induction; LTR, low-temperature-responsive element; MBS and TC-rich repeats, involved in defense and stress responses; G-box and GT1 motif, light-responsive elements; CAT box and GC motif, involved in meristem expression and anoxic-specific inducibility, respectively.

To investigate the regulatory mechanisms of *DrSWEET* genes, we performed a cis-regulatory element prediction analysis. Among the *DrSWEET* genes, *DrSWEET19* contained the highest number of binding sites associated with gibberellin biosynthesis regulation. *DrSWEET14* and *DrSWEET*15 had binding sites involved in flavonoid biosynthesis regulation, while only *DrSWEET*19 contained an HD-Zip 1 element, which is associated with mesophyll cell differentiation. Notably, cis-acting elements related to circadian rhythm regulation were found exclusively in *DrSWEET*11 and *DrSWEET*12. We analyzed the cis-regulatory elements within the 2000 bp upstream regions of the *DrSWEET* genes (Figure 5A). The number of cis-elements varied significantly among genes, with *DrSWEET4* containing the highest number (47 elements) and *DrSWEET8* containing the lowest number (18 elements). A high frequency of light-responsive elements was observed in the promoter regions of many *DrSWEET* genes, suggesting their regulation by light signaling pathways. In addition, hormone-responsive elements were widely present, including the CGTCA motif (18.45%) and TCA element (11.65%), which are associated with hormonal signaling. Elements related to light responses included Box 4 (48.60%) and G-Box (23.36%). Stress-related cis-elements were also abundant in *DrSWEET* promoters, including ABRE (45.63%) for stress responses, ARE (15.05%) for anaerobic induction, MYC (38.83%) and MYB (33.50%) for drought responses, LTR (3.88%) for cold stress, and CAT-box (3.40%) for meristem regulation. To further explore the functional regulation of *DrSWEET* genes, we visualized key hormone-responsive, growth-related, and stress-related elements (Figure 5B). The results revealed that the majority of *DrSWEET* promoters contained hormone-related elements, including MeJA (*n* = 38), ABA (*n* = 47), SA (*n* = 12), and IAA (*n* = 6). Additionally, stress-related cis-elements involved in anaerobic induction (*n* = 31), drought responses (*n* = 58), and cold stress responses (*n* = 8) were identified. Moreover, elements related to plant growth and development regulation, such as light responsiveness (*n* = 214) and meristem expression (*n* = 7), were detected. These findings suggest that *DrSWEET* genes are likely regulated by multiple cis-elements, influencing both growth and stress responses. The presence of multiple cis-regulatory elements in most *DrSWEET* promoters implies their involvement in diverse stress response networks, suggesting distinct and complex regulatory mechanisms. Furthermore, within the same *SWEET* gene group, the variation in the type and number of stress- and hormone-responsive elements indicates gene-specific and context-dependent expression patterns (Figure 5B).

### 2.7. Expression Patterns of DrSWEET Genes in Different Tissues

**Figure 6:**
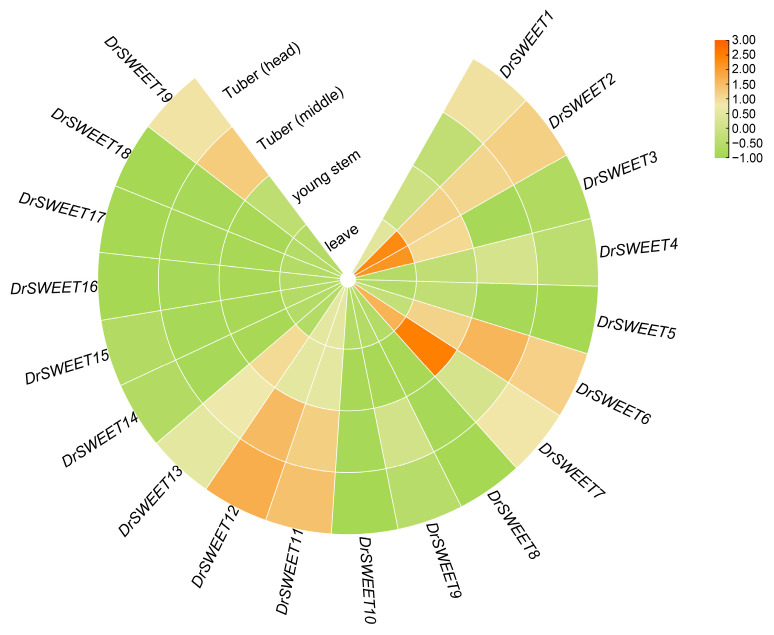
Expression profiles of the *19 DrSWEET* genes across five tissues of *Dioscorea rotundata*, including leaf, young stem, tuber (middle), and tuber (head). Transcript abundance was quantified using RNA-seq and is presented as log_2_-transformed FPKM values. Genes with log_2_(FPKM) > 1.5 were considered to exhibit elevated expression. The radial heatmap displays relative expression levels, with green indicating low expression and orange indicating high expression.

It illustrates the expression profiles of 19 *DrSWEET* genes across five tissues in *Dioscorea rotundata,* based on log_2_-transformed FPKM values. Genes with log_2_(FPKM) > 1.5 were considered to have elevated expression. Several members, including *DrSWEET1, DrSWEET2, DrSWEET6, DrSWEET7, DrSWEET11,* and *DrSWEET12*, showed relatively high transcript abundance in leaves and young stems, suggesting roles in early-stage sugar transport. Among these, *DrSWEET1* and *DrSWEET2* maintained consistently high expression across all tissues.*DrSWEET4* was highly expressed in leaves, possibly involved in sugar efflux. *DrSWEET13* and *DrSWEET14* were predominantly active in young stems and tubers, implying tissue-specific functions in sink organs. In contrast, several genes, including *DrSWEET9, DrSWEET10*, and *DrSWEET19*, displayed uniformly low expression, suggesting restricted or condition-dependent activity. These patterns indicate functional divergence within the *DrSWEET* family, with certain genes exhibiting tissue-specific roles in sugar allocation during plant development (Figure 6).

### 2.8. Expression Patterns of DrSWEET Genes in Tubers at Different Development Stages

**Figure 7:**
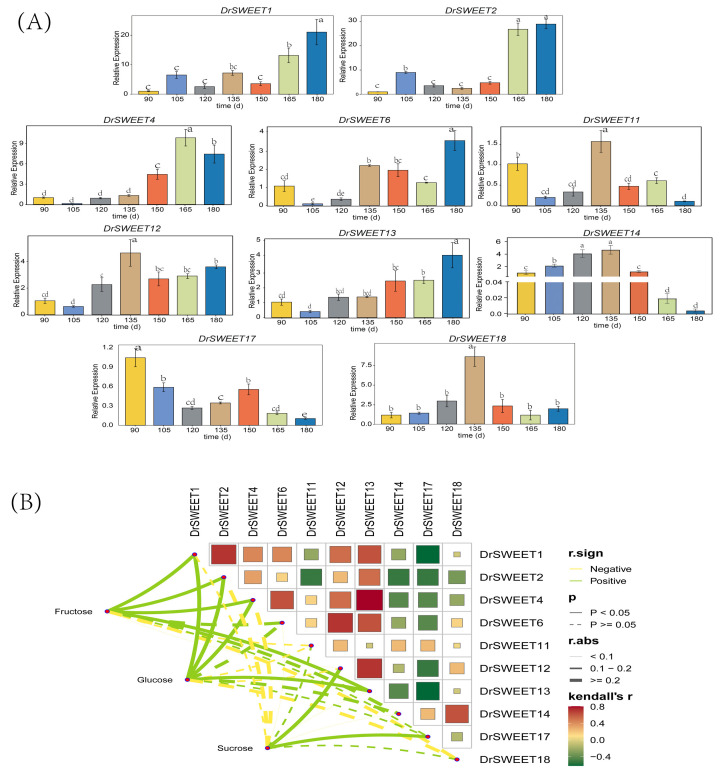
(**A**) The relative expression levels of *DrSWEET* genes in different developmental phases. Different lowercase letters on the bar indicate significant differences among treatments (*p* < 0.05). (**B**) The matrix and network diagram illustrate Kendall’s correlation coefficients (r) between the expression levels of *DrSWEET* genes and the concentrations of fructose, glucose, and sucrose across sampling stages. In the matrix (upper right), color indicates correlation direction and strength (green: positive; red: negative), while square size reflects correlation magnitude. In the network (bottom left), edge color represents correlation direction (green: positive; yellow: negative), line type indicates significance (solid: *p* < 0.05; dashed: *p* ≥ 0.05), and line width corresponds to correlation strength.

The expression of *DrSWEET*11, *DrSWEET*12, *DrSWEET*14, and *DrSWEET*18 during tuber development shows an initial increase followed by a decline, indicating the roles of these genes in both the initiation and decline phases. *DrSWEET*12 and *DrSWEET*14 peak in early to mid-development, suggesting regulatory functions in sugar translocation for tuber formation. The expression of *DrSWEET*13 steadily increases, supporting continuous growth, while that of *DrSWEET*17 decreases, implying an early-stage role. The remaining nine *SWEET* family members show no expression in tubers, reflecting tissue-specific patterns, likely functioning in other tissues, such as leaves, roots, and reproductive organs, for sugar transport or other processes (Figure 7A). To investigate the potential regulatory relationships between soluble carbohydrates and the *SWEET* gene family, a Kendall’s correlation analysis was performed between the concentrations of fructose, glucose, and sucrose and the expression levels of *DrSWEET* genes across different sampling stages (Figure 7B). The analysis revealed that *DrSWEET1*, *DrSWEET2*, and *DrSWEET4* exhibited significant positive correlations with both fructose and glucose levels (*p* < 0.05), suggesting their involvement in monosaccharide transport or associated regulatory pathways. Notably, *DrSWEET12* displayed a strong and significant positive correlation with sucrose (|r| > 0.2, *p* < 0.05) and a moderate positive trend with fructose, implying a pivotal role in sucrose translocation and possibly broader carbohydrate metabolism. *DrSWEET17* also showed a significant positive correlation with sucrose, further supporting its potential function in sucrose partitioning. In contrast, *DrSWEET6* was significantly negatively correlated with both fructose and glucose, particularly with glucose (*p* < 0.05), indicating a potential repressive role in sugar accumulation or transport. This suggests that *DrSWEET6* and *DrSWEET12* may exert opposing regulatory effects in carbohydrate dynamics. Additionally, *DrSWEET13* was negatively correlated with both fructose and glucose with relatively high coefficients, highlighting its potential role in negative regulation. While *DrSWEET14* and *DrSWEET18* demonstrated generally positive correlations with all three sugars, most did not reach statistical significance, warranting further functional validation. Collectively, these results reveal a complex and differential association between *DrSWEET* gene expression and sugar levels, emphasizing the potential central roles of *DrSWEET6* and *DrSWEET12* in the regulation of carbohydrate metabolism in *Dioscorea rotundata*.

### 2.9. Subcellular Localization of DrSWEET Proteins

**Figure 8:**
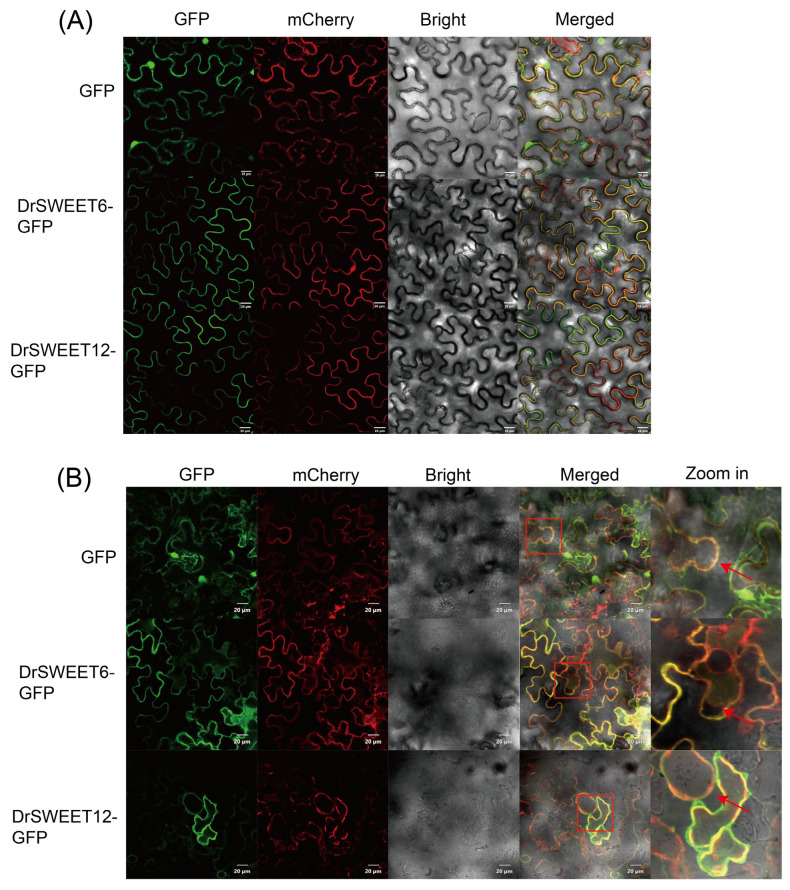
(**A**) Subcellular localization of two representative SWEET proteins. GFP is indicated as empty in the figure, and a membrane localization protein (pCAMBIA1300-35S-PM-mCherry) tagged using co-transformed mCherry was used to visualize the plasma membranes. (**B**) Subcellular localization analysis was also conducted in plasmolyzed tobacco cells to further verify the precise localization of the proteins between the plasma membrane and the cytoplasm. The fields included the green fluorescence field (488 nm), nucleus autofluorescence field (570 nm), bright field, and merged field. The red square highlights the region shown at higher magnification, and the red arrow indicates the position of the plasma membrane. Scale bar = 20 μm.

To determine the subcellular localization of DrSWEET proteins, two representative genes, DrSWEET12 and DrSWEET6, were selected to construct GFP fusion proteins (DrSWEET12-GFP and DrSWEET6-GFP), which were cloned into the pCAMBIA1302-GFP vector and expressed under the control of the CaMV 35S promoter. The selection of these genes was based on the following criteria: both exhibit the highest and most stable expression levels across all tuber bulking stages (90–180 DAP) in the RNA-seq data; they belong to two evolutionarily distinct clades (clades I and III), thus representing structural diversity; and they share high sequence similarity with known sucrose transporters, such as AtSWEET9–AtSWEET15, making them strong candidates for functional validation. The fusion constructs were transiently expressed in Nicotiana benthamiana epidermal cells and co-expressed with the plasma membrane marker pCAMBIA1300-35S-PM-mCherry (Figure 8A). To further verify membrane localization, after treating the tobacco leaf samples with 30% sucrose solution for 5 min to induce plasmolysis, we performed confocal microscopy to examine the subcellular localization of the GFP fusion proteins. The GFP fluorescence signal remained tightly associated with the plasma membrane marker (pCAMBIA1300-35S-PM-mCherry) even after plasmolysis, showing strong co-localization without any noticeable displacement into the cytoplasm. Notably, in the magnified regions highlighted by red boxes (Zoom in), the GFP and mCherry signals precisely overlap along the plasma membrane contour. These results provide further evidence that the target proteins are localized to the plasma membrane and effectively exclude the possibility of cytosolic or organellar localization (Figure 8B).

### 2.10. Transport Substrate Specificity of DrSWEET6 and DrSWEET12 in Yeast

**Figure 9:**
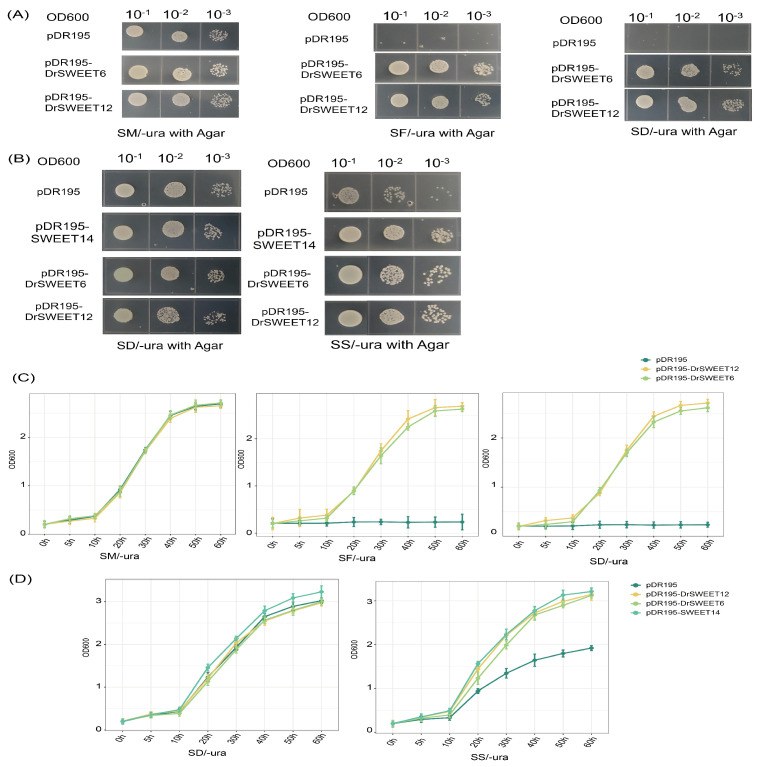
Analysis of the transport activity of DrSWEET6 and DrSWEET12 in yeast cells. After adjusting the OD600 value of the above positive strains to 2, 2.5 µL samples were taken and subjected to serial dilution for plating at 10^−1^, 10^−2^, and 10^−3^ dilutions. (**A**) Growth of the yeast mutant strain EBY.VW4000 expressing different genes in SD (-Ura) media supplemented with various carbon sources: 2% maltose (SM/-ura with agar), 2% fructose (SF/-ura with agar), or 2% glucose (SD/-ura with agar). Yeast strains transformed with the pDR195 empty vector were used as negative controls. (**B**) Growth of the yeast mutant strain SUSY7/ura3 expressing different genes in SD (-Ura) media supplemented with 2% glucose or 2% sucrose (SS/-ura with agar). The pDR195 empty vector was used as a negative control, and AtSUC2 was included as a positive control. Yeast cells of the EBY.VW4000 strain were grown at 30 °C for 3–5 days, while those of the SUSY7/ura3 strain were grown at 30 °C for 3–5 days. (**C**) Growth of EBY.VW4000 (hexose transporter-deficient) expressing DrSWEET6, DrSWEET12, or empty vector (pDR195) in media containing glucose (SM-ura), fructose (SF-ura), or maltose (SD-ura). OD600 was measured over time. (**D**) Growth of SUSY7/ura3 (sucrose uptake-deficient) expressing DrSWEET6, DrSWEET12, SWEET14 (positive control), or pDR195 in SD-ura (negative control) and SS-ura (sucrose medium). OD600 was recorded to assess sucrose uptake. Error bars represent standard deviation (n = 3).

In this study, the ability of two DrSWEET proteins to transport hexoses and sucrose was investigated through heterologous expression of their cDNAs in the hexose transporter-deficient yeast strain EBY.VW4000 and the sucrose uptake-deficient strain SUSY7/ura3. The latter lacks extracellular invertase and thus cannot utilize external sucrose as the sole carbon source, but exhibits sucrose synthase activity that enables metabolism of sucrose transported by exogenous transporters (Figure 9A). The open reading frames of DrSWEET6 and DrSWEET12 were cloned into the yeast expression vector pDR195 to generate pDR195-DrSWEET6 and pDR195-DrSWEET12. Expression of these constructs restored the growth of EBY.VW4000 in media supplemented with glucose or fructose (Figure 9B). Similarly, SUSY7/ura3 cells expressing DrSWEET6, DrSWEET12, or the positive control AtSWEET14 grew faster than control cells in sucrose-only medium, indicating their ability to mediate sucrose uptake. To further characterize substrate specificity, growth curve analyses were performed. In glucose (SM-ura), fructose (SF-ura), and maltose (SD-ura) media EBY.VW4000 cells expressing DrSWEET6 or DrSWEET12 showed markedly enhanced growth compared to the empty vector control, confirming their hexose transport capability (Figure 9C). In sucrose-supplemented media (SS-ura), SUSY7/ura3 cells expressing DrSWEET6, DrSWEET12, or AtSWEET14 also exhibited significantly increased growth relative to the control (Figure 9D), with growth kinetics comparable to the positive control, indicating efficient sucrose transport. These quantitative growth analyses, together with complementation assays, demonstrate that DrSWEET6 and DrSWEET12 can transport both mono- and disaccharides, highlighting their dual substrate specificity and reinforcing the functional conservation of SWEET transporters across different clades.

## 3. Discussion

Carbohydrates are exported by the SWEET transporter family, a novel class of sugar transporters that mediate transmembrane movement and facilitate long-distance translocation from source to sink tissues. Sugars play pivotal roles in regulating diverse physiological processes by modulating apoplastic and symplastic sugar concentrations. A phylogenetic analysis of *Dioscorea rotundata SWEET* (*DrSWEET*) genes revealed four major clades (I–IV), consistent with the classification established in *Arabidopsis thaliana. SWEET* genes have been identified across a range of plant species, including 21 in rice (*Oryza sativa*) [20], 105 in wheat (*Triticum aestivum* L.) [21], 23 in sorghum (*Sorghum bicolor*) [22], and 52 in soybean (*Glycine max*) [23]. The expansion of *SWEET* gene family members across plant lineages suggests functional diversification to accommodate species-specific physiological needs. A subcellular localization analysis indicated that most DrSWEET proteins are localized to the plasma membrane and that all members possess the conserved MtN3/saliva domain, underscoring the evolutionary conservation of this gene family [24].Based on phylogenetic analyses, the SWEET proteins in plants are generally classified into four clades [25]. In *Arabidopsis*, clades I and II primarily mediate hexose transport. For instance, *OsSWEET5* and *AtSWEET1* are responsible for transporting galactose and glucose across the plasma membrane, respectively [6,26]. Clade III members are predominantly sucrose transporters, facilitating sucrose efflux from phloem parenchyma cells into the apoplast, enabling phloem loading and long-distance transport [1]. *DrSWEET6* and *DrSWEET12*, two plasma membrane-localized SWEET transporters identified in yam, have been shown to mediate the transport of both hexoses and sucrose. SWEET proteins are recognized as bidirectional transporters that facilitate both the cellular uptake and efflux of sugars [6]; however, substrate specificity varies among SWEET family members [14].A phylogenetic analysis revealed that *DrSWEET6* and *DrSWEET12* belong to clades I and IV, respectively. Our results demonstrate that both transporters localize to the plasma membrane and are capable of transporting glucose, fructose, and sucrose. Similar observations have been made in other species; for example, *AcSWEET10* in clade II in pineapple functions as a glucose transporter [18], while *VvSWEET10* in clade III in grapevine has been characterized as a hexose-preferring transporter [27]. These findings suggest that the substrate specificity of SWEET proteins may be species-dependent and influenced by evolutionary divergence across different clades.

Gene duplication has played a fundamental role in the evolutionary diversification of the *SWEET* gene family. A comparative evolutionary analysis of Cucumis sativus showed that 96% of *SWEET* gene pairs have Ka/Ks ratios below 1, indicative of strong purifying selection [28]. This trend has also been observed in species such as Juglans, Capsicum annuum, and Helianthus annuus [29,30,31]. Similarly, *DrSWEET* genes appear to be under strong evolutionary constraint, suggesting the conservation of essential functions in sucrose transport. Notably, *DrSWEET3* exhibited a slightly elevated Ka/Ks ratio (Supplementary Table S2), which may reflect potential neofunctionalization or subfunctionalization, possibly related to enhanced sucrose unloading in tuber tissues under specific developmental or environmental signals. Further biochemical and transport assays are required to validate this hypothesis. The functional specificity of *DrSWEET* genes is closely linked to their transcriptional regulation, particularly in response to environmental stresses. A cis-element analysis of promoter regions revealed the presence of complex regulatory motifs associated with plant development and stress adaptation. For instance, *DrSWEET19* contained the highest number of gibberellin-responsive elements and an HD-Zip 1 element, implying a role in growth regulation [32]. *DrSWEET14* and *DrSWEET15* carried elements involved in flavonoid biosynthesis, while light- and hormone-responsive elements were broadly distributed, suggesting regulation by multiple signaling pathways [31,33]. Stress-related motifs, including MYC and MYB for drought, as well as LTR for cold responses, further support the involvement of *DrSWEET* genes in abiotic stress tolerance [8,34]. Collectively, these regulatory features indicate that *DrSWEET* genes are subject to multifaceted control by both environmental and endogenous signals [35]. Expression profiling across various tissues revealed complex regulatory dynamics during tuber development in *Dioscorea rotundata*. A cluster analysis of expression patterns suggested both coordinated regulation and functional divergence among *DrSWEET* members. *DrSWEET2*, *DrSWEET7*, *DrSWEET1*, *DrSWEET6*, *DrSWEET11*, and *DrSWEET12* were highly expressed in multiple tissues, particularly in leaves, indicating potential involvement in phloem loading and long-distance sugar transport from source to sink tissues. This observation aligns with previous findings in *Arabidopsis* showing that *AtSWEET11* and *AtSWEET12* facilitate sucrose efflux from source tissues [36]. Notably, *DrSWEET2* maintained consistently high expression across all tissues, suggesting a central role in maintaining sugar homeostasis. *DrSWEET11* and *DrSWEET12* showed elevated expression in tubers, supporting a role in sucrose unloading during starch accumulation and cell expansion—critical processes in tuber swelling. In contrast, *DrSWEET13* and *DrSWEET4* exhibited tissue-specific expression in young stems and tubers but were weakly expressed in leaves, indicating specialized roles in sink tissue sugar allocation. *DrSWEET3* was preferentially expressed in leaves and young stems, similar to *SlSWEET4* in tomato [37] and *OsSWEET1* in rice [38], suggesting functional conservation in sugar transport at the source end. Meanwhile, the low expression of some *DrSWEET* genes in storage organs may point to regulatory or signaling roles rather than direct sugar transport. Given that tubers serve as the primary sink organs in *Dioscorea rotundata,* the spatial and temporal expression dynamics of *DrSWEET* genes underscore their importance in coordinating sugar distribution and developmental progression. These results lay the groundwork for future functional analyses of individual SWEET transporters in yam, especially regarding their roles in carbohydrate metabolism and environmental adaptation [39]. Furthermore, the elevated expression of several *DrSWEET* genes during mid-tuber development likely corresponds to the peak phase of tuber expansion. This stage is characterized by intense cell division and enlargement, necessitating enhanced sugar transport and carbohydrate accumulation. The upregulation of *DrSWEET12*, *DrSWEET13*, and *DrSWEET14* highlights their involvement in meeting energy demands and supporting biomass accumulation and structural development during this critical period [40]. These dynamic expression patterns emphasize the stage-specific and functionally diverse roles of *DrSWEET* genes in tuber development.

## 4. Materials and Methods

### 4.1. Plant Materials

An experiment was conducted with yam, cultivated in sandy loam soil at the experimental site of Inner Mongolia Agricultural University during a recent growing season. A complete block design was used for planting, with each row containing 80–90 plants and a spacing of 80 cm between rows. To investigate the developmental progression of yam, samples were collected from various plant tissues, including the head and middle sections of tubers, stems, and leaves, at several growth stages: 90 days (early tuber initiation), 105 days (early tuber bulking), 120 days (mid tuber bulking), 135 days (peak tuber bulking), 150 days (late tuber bulking), 165 days (end of tuber bulking), and 180 days (maturity) after planting. For each sampling time point, three biological replicates were chosen for RNA extraction. The tuber samples collected at 90 days were immediately flash-frozen in liquid nitrogen and stored at −80 °C for subsequent analysis.

### 4.2. Identification of the SWEET Genes in Yams

We downloaded the whole-genome and proteome data of “*Dioscorea zingiberensis*“, “*Dioscorea alata*”, and “*Dioscorea rotundata*” from the Yam Omics database (https://biotec.njau.edu.cn/yamdb/, accessed on 18 December 2024) [19]. The SWEET protein sequences of *Dioscorea rotundata* were obtained from NCBI (https://www.ncbi.nlm.nih.gov/datasets/genome/GCF_009730915.1/, accessed on 18 December 2024), and the AtSWEET protein sequences were obtained from the Arabidopsis Information Resource (TAIR, version 10, http://www.arabidopsis.org, accessed on 18 December 2024) [41]. A local protein database was created, and BLASTP (E-value < 1 × 10^−5^) was used to identify potential SWEET family members by aligning the sequences. In addition, the HMM file for the SWEET domain (PF13347) was retrieved from the Pfam database (http://pfam-legacy.xfam.org/, accessed on 18 December 2024) [42], and HMMER v3.3.2 software was applied to detect possible SWEET proteins [43]. Candidate sequences were further verified by submitting them to SMART (http://smart.embl-heidelberg.de/, accessed on 18 December 2024) [44], and to ensure the integrity of the SWEET domain, we compared the identified SWEET family members using tools from the NCBI CDD (https://www.ncbi.nlm.nih.gov/cdd, accessed on 18 December 2024) [45]. To predict the molecular characteristics of the SWEET proteins, including length, molecular weight, theoretical isoelectric point, instability coefficient, hydrophobicity, and average hydrophilicity, we used the ProtParam tool (https://web.expasy.org/protparam/, accessed on 18 December 2024) [46]. Additionally, the transmembrane structure, subcellular localization, and secondary structure of the SWEET family members in *Dioscorea rotundata* were predicted using TMHMM2.0 [47] (https://services.healthtech.dtu.dk/services/TMHMM-2.0/, accessed on 18 December 2024) Cell-PLoc (http://www.csbio.sjtu.edu.cn/bioinf/Cell-PLoc-2/, accessed on 18 December 2024) and SOPMA (https://npsa-prabi.ibcp.fr/cgi-bin/npsa_automat.pl?page=npsa_sopma.html, accessed on 18 December 2024).

### 4.3. Multiple Sequence Alignment and Phylogenetic Analysis of SWEET Genes

To investigate the phylogenetic relationships of *SWEET* genes, the SWEET protein sequences of *Arabidopsis thaliana* and *Dioscorea rotundata* were retrieved from the UniProt database (https://www.uniprot.org, accessed on 18 December 2024) for the construction of a neighbor-joining (NJ) phylogenetic tree. The SWEET protein sequences of all plant species were aligned using ClustalX 1.81. A phylogenetic tree, including multiple plant species (*Arabidopsis thaliana* and *Dioscorea rotundata*), was constructed by using Mega11.0 with the NJ method, selecting the Poisson model, and performing 1000 bootstrap replications for validation. Prior to tree construction, we aligned the amino acid sequences of the *DrSWEET* and *AtSWEET* genes using ClustalX 1.81.(Supplementary Table S3). The DNA and cDNA sequences of the *DrSWEET* gene were used to predict intron structures via the online Gene Structure Display Server (GSDS) 2.0 (http://gsds.gao-lab.org/, accessed on 18 December 2024). The conserved motifs of the SWEET proteins in various plant species were identified using MEME software (http://meme.nbcr.net/meme/intro.html, accessed on 18 December 2024) [48].

### 4.4. Chromosomal Mapping, Gene Replication, and Syntenic Analysis with Other Plant Species

The physical locations of the *DrSWEET* genes were obtained from the genome annotation file downloaded from the Yam Genomics Database and visualized with Tbtools. The collinear relationships of the *DrSWEET* genes were analyzed using Dual Synteny Plotter software (https://github.com/CJ-Chen/TBtools-II, accessed on 18 December 2024). The tandem replication and segmental replication events in the *DrSWEET* genes were analyzed using multiple collinear scanning toolkits (MCScanX). All *DrSWEET* genes were found to be located on the 8 chromosomes of *Dioscorea rotundata*. The SWEET collinearity pairs between “*Dioscorea* zingiberensis”, “*Dioscorea* alata”, and “*Dioscorea rotundata*” were extracted in Tbtools and used for collinearity mapping [49].

### 4.5. Identification of Cis-Regulatory Elements in the Promoter Regions of Pear SWEET Genes

To allow for the identification of cis-regulatory elements in the promoter regions of *SWEET* genes, TBtools was used to extract the DNA sequence 2000 bp upstream of the *SWEET* promoter region in the yam genome. These genes were submitted to the PlantCARE database (http://bioinformatics.psb.ugent.be/webtools/plantcare/html/, accessed on 28 April 2024) [50]. Cis-acting elements were identified, and stress response, plant growth and development, and hormone response elements were screened.

### 4.6. Expression of DrSWEET Genes According to Quantitative Real-Time Polymerase Chain Reaction (qRT-PCR)

The qRT-PCR primers for the *DrSWEET* genes were designed using Primer 5 (https://www.premierbiosoft.com/index.html, accessed on 28 April 2024), and the specific primer information is provided in (Supplementary Table S4),. In this experiment, the internal reference gene used was UBQ [36]. The primers used in our experiments were synthesized by Sangon Biotech (Shanghai) Co., Ltd. (Shanghai, China) The company’s website is:https://www.sangon.com. qRT-PCR analysis was conducted using SYBR® Premix ExTaq™ II (Tli RNaseH Plus, RR820A; TaKaRa Biotechnology, Dalian, China) on an FTC-3000P system (Funglyn Biotech, Toronto, ON, Canada), according to the manufacturer’s Gene expression data were calculated using the 2^−ΔΔCT^ method. The experiments were conducted with three biological replicates, and three technical replicates were performed for each biological replicate [51].

### 4.7. Transcriptome Data Analysis

A transcriptome data analysis was conducted based on publicly available databases. Transcriptome datasets of yam (*Dioscorea rotundata*) corresponding to different tissue types—including the young stem, mature stem, middle tuber, and tuber apex—were retrieved from the NCBI Sequence Read Archive under project PRJDB3383 (https://www.ncbi.nlm.nih.gov/sra/?term=DRR063126). The aim was to investigate gene expression patterns in distinct yam tissues. The OmicStudio platform (Maiwei Cloud) was employed to generate heatmaps for the visualization of differential gene expression levels. The quantification of sucrose, glucose, and fructose in yam tubers was carried out according to established physiological methods described in previous studies [52].

### 4.8. Yeast Complementation Assay

A yeast complementation assay was carried out using the hexose transport-deficient yeast strain EBY.VW4000 and the mutant strain SUSY7/ura3, both purchased from PyPoint (https://www.pytbio.com/). To perform the complementation experiments in Saccharomyces cerevisiae cells, the orfs of two *SWEET* genes, each containing XhoI and BamHI restriction sites, were cloned into the yeast expression vector pDR195. The recombinant plasmids pDR195-DrSWEET6 and pDR195-DrSWEET12, the empty vector pDR195, and the positive control vector pDR195-SWEET14 were transformed into competent cells of EBY.VW4000 and SUSY7/ura3. The transformation products of EBY.VW4000 were plated on SM/-Ura agar plates, while those of SUSY7/ura3 were plated on SD/-Ura agar plates. Positive clones obtained from the EBY.VW4000 competent cells were resuspended in sterile saline and adjusted to an OD600 of 0.2. These cells were then serially diluted by factors of 100 and 1000, and 2.5 µL of each dilution was spotted onto SM/-Ura agar plates (supplemented with maltose), SF/-Ura agar plates (supplemented with fructose), and SD/-Ura agar plates (supplemented with glucose), followed by incubation at 30 °C for 3–5 days. Positive clones obtained from SUSY7/ura3 competent cells were similarly resuspended in sterile saline, adjusted to an OD600 of 0.2, and 2.5 µL of each dilution was spotted onto SD/-Ura agar plates (supplemented with glucose) and SS/-Ura agar plates (supplemented with sucrose), with incubation at 30 °C for 3–5 days.

## 5. Conclusions

Despite its nutritional and agricultural importance, molecular studies on yam are limited, with most focusing on nutritional composition analysis. In particular, the mechanisms of carbohydrate transport during tuber development, especially those involving sucrose translocation, remain poorly understood. While previous studies in other crops have largely focused on gene identification and expression profiling, our study integrates bioinformatics analysis and functional assays to uncover conserved substrate-binding features in yam SWEET proteins, offering novel insights into transporter engineering. Given the key role of sucrose transporters in biomass accumulation and sink organ development, we performed a genome-wide analysis of the *SWEET* gene family in yam, including phylogenetic relationships, gene structures, and expression profiles across developmental stages. Furthermore, we identified two SWEET proteins, *DrSWEET6* and *DrSWEET12*, with expression patterns linked to key stages of tuber development, and validated their substrate specificity through yeast uptake assays. Growth assays in yeast mutant strains further demonstrated that these transporters affect sucrose-dependent growth, confirming their physiological relevance. Together, our results bridge genomic, bioinformatics, and functional analyses to provide new insights into SWEET-mediated carbohydrate transport in yam. This work not only enhances the current understanding of tuber sink strength and sugar allocation but also lays the groundwork for molecular breeding strategies to improve yield and quality in yam and other tuber crops.

## Figures and Tables

**Table 1:** The characteristics of the *DrSWEET* gene family.

ID	Number of Amino Acids	Molecular Weight	Theoretical pI	Instability Index	Aliphatic Index	Grand Average of Hydropathicity	Subcellular Localization
DrSWEET1	265	30,100.93	8.84	34.82	116.15	0.728	PlasmaMembrane
DrSWEET2	259	28,879.07	8.87	36.92	107.61	0.55	PlasmaMembrane
DrSWEET3	249	27,792.97	8.59	45.54	116.67	0.646	PlasmaMembrane
DrSWEET4	231	25,941.74	9.05	36.57	123.55	0.824	PlasmaMembrane
DrSWEET5	278	31,401.43	8.71	30.48	116.01	0.655	PlasmaMembrane
DrSWEET6	292	32,414.34	9.63	39.47	109.76	0.401	PlasmaMembrane
DrSWEET7	274	30,542.61	8.78	33.73	126.28	0.726	PlasmaMembrane
DrSWEET8	260	28,541.97	9.01	27.58	116.88	0.754	PlasmaMembrane
DrSWEET9	153	17,072.41	6.39	49.59	130	0.666	PlasmaMembrane
DrSWEET10	151	16,772.87	5.58	37.26	109.67	0.695	PlasmaMembrane
DrSWEET11	237	26,533.57	9.49	39.49	121.31	0.916	PlasmaMembrane
DrSWEET12	215	23,990.57	9.46	41.9	120.14	0.927	PlasmaMembrane
DrSWEET13	246	27,264.68	9.3	38.95	122.4	0.652	PlasmaMembrane
DrSWEET14	300	33,682.69	9.56	27.54	131.2	0.859	PlasmaMembrane
DrSWEET15	248	27,944.52	9.61	28.89	126.9	0.667	PlasmaMembrane
DrSWEET16	237	26,257.78	9.25	40.75	128.61	0.84	PlasmaMembrane
DrSWEET17	237	26,821.32	9.04	38.6	115.11	0.757	PlasmaMembrane
DrSWEET18	238	26,763.48	9.04	43.35	128.53	0.83	PlasmaMembrane
DrSWEET19	254	28,296.95	9	30.54	119.29	0.771	PlasmaMembrane

## Data Availability

Data will be made available on request.

## Supplementary Materials

The following supporting information can be downloaded at: https://www.mdpi.com/article/10.3390/ijms26125847/s1.

## References

[1] Chen L.-Q., Qu X.-Q., Hou B.-H., Sosso D., Osorio S., Fernie A.R., Frommer W.B. (2012). Sucrose efflux mediated by SWEET proteins as a key step for phloem transport. Science.

[2] Comtet J., Turgeon R., Stroock A.D. (2017). Phloem Loading through Plasmodesmata: A Biophysical Analysis. Plant Physiol..

[3] Liu W., Jiang H., Zeng F. (2025). The sugar transporter proteins in plants: An elaborate and widespread regulation network—A review. Int. J. Biol. Macromol..

[4] Wang Z., Wei X., Yang J., Li H., Ma B., Zhang K., Zhang Y., Cheng L., Ma F., Li M. (2020). Heterologous expression of the apple hexose transporter MdHT_2.2_ altered sugar concentration with increasing cell wall invertase activity in tomato fruit. Plant Biotechnol. J..

[5] Gibson S.I. (2005). Control of plant development and gene expression by sugar signaling. Curr. Opin. Plant Biol..

[6] Chen L.-Q., Hou B.-H., Lalonde S., Takanaga H., Hartung M.L., Qu X.-Q., Guo W.-J., Kim J.-G., Underwood W., Chaudhuri B. (2010). Sugar transporters for intercellular exchange and nutrition of pathogens. Nature.

[7] Yuan M., Wang S. (2013). Rice MtN3/Saliva/SWEET Family Genes and Their Homologs in Cellular Organisms. Mol. Plant.

[8] Gautam T., Dutta M., Jaiswal V., Zinta G., Gahlaut V., Kumar S. (2022). Emerging Roles of SWEET Sugar Transporters in Plant Development and Abiotic Stress Responses. Cells.

[9] Guan Y.-F., Huang X.-Y., Zhu J., Gao J.-F., Zhang H.-X., Yang Z.-N. (2008). *RUPTURED POLLEN GRAIN1*, a Member of the MtN3/saliva Gene Family, Is Crucial for Exine Pattern Formation and Cell Integrity of Microspores in Arabidopsis. Plant Physiol..

[10] Wang J., Xue X., Zeng H., Li J., Chen L.-Q. (2022). Sucrose rather than GA transported by AtSWEET13 and AtSWEET14 supports pollen fitness at late anther development stages. New Phytol..

[11] Eom J.-S., Chen L.-Q., Sosso D., Julius B.T., Lin I.W., Qu X.-Q., Braun D.M., Frommer W.B. (2015). SWEETs, transporters for intracellular and intercellular sugar translocation. Curr. Opin. Plant Biol..

[12] Huang C., Yu J., Cai Q., Chen Y., Li Y., Ren Y., Miao Y. (2020). Triple-localized WHIRLY2 Influences Leaf Senescence and Silique Development via Carbon Allocation. Plant Physiol..

[13] Singh J., Das S., Jagadis Gupta K., Ranjan A., Foyer C.H., Thakur J.K. (2023). Physiological implications of SWEETs in plants and their potential applications in improving source-sink relationships for enhanced yield. Plant Biotechnol. J..

[14] Jeena G.S., Kumar S., Shukla R.K. (2019). Structure, evolution and diverse physiological roles of SWEET sugar transporters in plants. Plant Mol. Biol..

[15] Breia R., Conde A., Badim H., Fortes A.M., Gerós H., Granell A. (2021). Plant SWEETs: From sugar transport to plant-pathogen interaction and more unexpected physiological roles. Plant Physiol..

[16] Zhang S., Wang H., Wang T., Zhang J., Liu W., Fang H., Zhang Z., Peng F., Chen X., Wang N. (2023). Abscisic acid and regulation of the sugar transporter gene MdSWEET9b promote apple sugar accumulation. Plant Physiol..

[17] Wu Y., Di T., Wu Z., Peng J., Wang J., Zhang K., He M., Li N., Hao X., Fang W. (2024). CsLHY positively regulates cold tolerance by activating CsSWEET17 in tea plants. Plant Physiol. Biochem..

[18] Fakher B., Ashraf M.A., Wang L., Wang X., Zheng P., Aslam M., Qin Y. (2023). Pineapple SWEET10 is a glucose transporter. Hortic Res.

[19] Chen Y., Tariq H., Shen D., Liu J., Dou D. (2024). Omics technologies accelerating research progress in yams. Veg. Res..

[20] Yuan M., Zhao J., Huang R., Li X., Xiao J., Wang S. (2014). Rice *MtN3/saliva/SWEET* gene family: Evolution, expression profiling, and sugar transport. J. Integr. Plant Biol..

[21] Qin J.-X., Jiang Y.-J., Lu Y.-Z., Zhao P., Wu B.-J., Li H.-X., Wang Y., Xu S.-B., Sun Q.-X., Liu Z.-S. (2020). Genome-wide identification and transcriptome profiling reveal great expansion of SWEET gene family and their wide-spread responses to abiotic stress in wheat (*Triticum aestivum* L.). J. Integr. Agric..

[22] Mizuno H., Kasuga S., Kawahigashi H. (2016). The sorghum SWEET gene family: Stem sucrose accumulation as revealed through transcriptome profiling. Biotechnol. Biofuels.

[23] Patil G., Valliyodan B., Deshmukh R., Prince S., Nicander B., Zhao M., Sonah H., Song L., Lin L., Chaudhary J. (2015). Soybean (*Glycine max*) SWEET gene family: Insights through comparative genomics, transcriptome profiling and whole genome re-sequence analysis. BMC Genom..

[24] Heng S., He J., Zhu X., Cai J., Fu M., Zhang S., Zeng W., Xing F., Mao G. (2024). Genome wide identification of BjSWEET gene family and drought response analysis of BjSWEET12 and BjSWEET17 genes in Brassica juncea. BMC Plant Biol..

[25] Yamada K., Osakabe Y. (2018). Sugar compartmentation as an environmental stress adaptation strategy in plants. Semin. Cell Dev. Biol..

[26] Zhou Y., Liu L., Huang W., Yuan M., Zhou F., Li X., Lin Y. (2014). Overexpression of OsSWEET5 in Rice Causes Growth Retardation and Precocious Senescence. PLoS ONE.

[27] Zhang Z., Zou L., Ren C., Ren F., Wang Y., Fan P., Li S., Liang Z. (2019). VvSWEET10 Mediates Sugar Accumulation in Grapes. Genes.

[28] Chen X., Wang Z., Tang R., Wang L., Chen C., Ren Z. (2021). Genome-Wide Identification and Expression Analysis of Hsf and Hsp Gene Families in Cucumber (*Cucumis sativus* L.). Plant Growth Regul..

[29] Ceylan Y., Altunoglu Y.C., Horuz E. (2023). HSF and Hsp Gene Families in sunflower: A comprehensive genome-wide determination survey and expression patterns under abiotic stress conditions. Protoplasma.

[30] Wu D., Luo J., Chen J., Zhang L., Lim K.J., Wang Z. (2019). Selection pressure causes differentiation of the SPL gene family in the Juglandaceae. Mol. Genet. Genom..

[31] Han X., Han S., Zhu Y., Liu Y., Gao S., Yin J., Wang F., Yao M. (2023). Genome-Wide Identification and Expression Analysis of the SWEET Gene Family in *Capsicum annuum* L. Int. J. Mol. Sci..

[32] Kanno Y., Oikawa T., Chiba Y., Ishimaru Y., Shimizu T., Sano N., Koshiba T., Kamiya Y., Ueda M., Seo M. (2016). AtSWEET13 and AtSWEET14 regulate gibberellin-mediated physiological processes. Nat. Commun..

[33] Cao L., Wang J., Wang L., Liu H., Wu W., Hou F., Liu Y., Gao Y., Cheng X., Li S. (2024). Genome-wide analysis of the SWEET gene family in *Hemerocallis citrina* and functional characterization of HcSWEET4a in response to salt stress. BMC Plant Biol..

[34] Klemens P.A.W., Patzke K., Deitmer J., Spinner L., Le Hir R., Bellini C., Bedu M., Chardon F., Krapp A., Neuhaus H.E. (2013). Overexpression of the Vacuolar Sugar Carrier *AtSWEET16* Modifies Germination, Growth, and Stress Tolerance in Arabidopsis. Plant Physiol..

[35] Kaur H., Manna M., Thakur T., Gautam V., Salvi P. (2021). Imperative role of sugar signaling and transport during drought stress responses in plants. Physiol. Plant..

[36] Durand M., Porcheron B., Hennion N., Maurousset L., Lemoine R., Pourtau N. (2016). Water Deficit Enhances C Export to the Roots in *Arabidopsis thaliana* Plants with Contribution of Sucrose Transporters in Both Shoot and Roots. Plant Physiol..

[37] Feng C.-Y., Han J.-X., Han X.-X., Jiang J. (2015). Genome-wide identification, phylogeny, and expression analysis of the SWEET gene family in tomato. Gene.

[38] Hu Z., Tang Z., Zhang Y., Niu L., Yang F., Zhang D., Hu Y. (2021). Rice SUT and SWEET Transporters. Int. J. Mol. Sci..

[39] Anjali A., Fatima U., Manu M.S., Ramasamy S., Senthil-Kumar M. (2020). Structure and regulation of SWEET transporters in plants: An update. Plant Physiol. Biochem..

[40] Chen L., Cai M., Liu J., Jiang X., Liu J., Zhenxing W., Wang Y., Li Y. (2024). Genome-wide identification and expression analyses of SWEET gene family reveal potential roles in plant development, fruit ripening and abiotic stress responses in cranberry (*Vaccinium macrocarpon* Ait). PeerJ.

[41] Reiser L., Bakker E., Subramaniam S., Chen X., Sawant S., Khosa K., Prithvi T., Berardini T.Z. (2024). The Arabidopsis Information Resource in 2024. Genetics.

[42] Li Y., Li D., Xiao Q., Wang H., Wen J., Tu J., Shen J., Fu T., Yi B. (2022). An in planta haploid induction system in Brassica napus. J. Integr. Plant Biol..

[43] Finn R.D., Clements J., Eddy S.R. (2011). HMMER web server: Interactive sequence similarity searching. Nucleic Acids Res..

[44] Letunic I., Khedkar S., Bork P. (2021). SMART: Recent updates, new developments and status in 2020. Nucleic Acids Res..

[45] Wang J., Chitsaz F., Derbyshire M.K., Gonzales N.R., Gwadz M., Lu S., Marchler G.H., Song J.S., Thanki N., Yamashita R.A. (2023). The conserved domain database in 2023. Nucleic Acids Res..

[46] Artimo P., Jonnalagedda M., Arnold K., Baratin D., Csardi G., de Castro E., Duvaud S., Flegel V., Fortier A., Gasteiger E. (2012). ExPASy: SIB bioinformatics resource portal. Nucleic Acids Res..

[47] Krogh A., Larsson B., von Heijne G., Sonnhammer E.L. (2001). Predicting transmembrane protein topology with a hidden Markov model: Application to complete genomes. J. Mol. Biol..

[48] Mistry J., Finn R.D., Eddy S.R., Bateman A., Punta M. (2013). Challenges in homology search: HMMER3 and convergent evolution of coiled-coil regions. Nucleic Acids Res..

[49] Chen C., Chen H., Zhang Y., Thomas H.R., Frank M.H., He Y., Xia R. (2020). TBtools: An Integrative Toolkit Developed for Interactive Analyses of Big Biological Data. Mol. Plant.

[50] Lescot M., Déhais P., Thijs G., Marchal K., Moreau Y., Van de Peer Y., Rouzé P., Rombauts S. (2002). PlantCARE, a database of plant cis-acting regulatory elements and a portal to tools for in silico analysis of promoter sequences. Nucleic Acids Res..

[51] Xing L., Zhang Y., Ge M., Zhao L., Huo X. (2024). Identification of WRKY gene family in Dioscorea opposita Thunb. reveals that DoWRKY71 enhanced the tolerance to cold and ABA stress. PeerJ.

[52] Maness N., Sunkar R. (2010). Extraction and Analysis of Soluble Carbohydrates. Plant Stress Tolerance: Methods and Protocols.

